# Characteristics of neonatal necrotizing enterocolitis in relation to the presence or absence of patent ductus arteriosus

**DOI:** 10.1186/s12884-025-07721-x

**Published:** 2025-06-02

**Authors:** Xin Lin, Lingling Liu, Huiping Zeng, Wenhong Cai

**Affiliations:** 1 Department of Neonatology, Fujian Maternity and Child Health Hospital/College of Clinical Medicine for Obstetrics & Gynecology and Pediatrics, Fujian Medical University, NO.18, Dao Shan Road, Fuzhou City, 350001 Fujian Province China; 2 Department of Electrophysiology, Fujian Maternity and Child Health Hospital/College of Clinical Medicine for Obstetrics & Gynecology and Pediatrics, Fujian Medical University, Fuzhou City, Fujian Province China; 3 Department of Pediatric Surgery, Fujian Maternity and Child Health Hospital/College of Clinical Medicine for Obstetrics & Gynecology and Pediatrics, Fujian Medical University, Fuzhou City, Fujian Province China; 4 Jinjiang Municipal Hospital (Shanghai Sixth People’s Hospital Fujian), Quanzhou City, Fujian Province China

**Keywords:** Patent Ductus Arteriosus, Necrotizing enterocolitis, Preterm Neonates, Echocardiography, Characteristics

## Abstract

**Objective:**

The presence of a patent ductus arteriosus (PDA) increases the morbidity and mortality rates in neonates with necrotizing enterocolitis (NEC), attributable to the "diastolic steal" phenomenon. Consequently, it is vital to assess the characteristics of NEC in relation to the presence or absence of PDA.

**Methods:**

A retrospective case–control study was conducted from 2015 to 2022, encompassing 135 neonates who were categorized into mild and severe NEC groups, with each group further divided into NO-PDA and PDA subgroups. Demographic characteristics of both mothers and neonates, as well as neonatal respiratory mechanics, hematologic parameters, and echocardiographic data were meticulously collected. Logistic regression analyses were conducted, and the results were statistically processed using the R programming language.

**Results:**

In the mild NEC group, 46.9% (38/81) of neonates had the presence of PDA, compared to 53.7% (29/54) in the severe NEC group. Additionally, a lower left ventricular systolic function and a higher incidence of moderate to severe tricuspid regurgitation were observed in the PDA subgroup of the severe NEC group (*P* < *0.05*). The occurrence of increased blood urea nitrogen, positive blood culture results in cases of sepsis, heart failure, and intraventricular hemorrhage was 1.27, 1.60, 1.91, and 3.86 times higher, respectively, in the PDA subgroup than in the NO-PDA subgroup. Within the PDA subgroup, for every one gram per liter decrease in fibrinogen, the incidence risk of NEC increased by 65%, and for each one mmol/L decrease in calcium levels, the incidence risk of NEC increased by 89% after adjustment.

**Conclusions:**

HsPDA in neonates not only affects the severity and progression of NEC but also exerts an impact on multi-system functions. This includes exacerbating respiratory issues (due to high pulmonary blood flow and increased need for ventilatory support), increasing the load on the left ventricular blood flow, promoting hypercoagulability, disrupting calcium metabolism, and raising the risk of sepsis and IVH.

**Supplementary Information:**

The online version contains supplementary material available at 10.1186/s12884-025-07721-x.

## Introduction

The ductus arteriosus (DA) is a physiological channel for blood flow between the pulmonary artery and the aorta. If the DA fails to close within 96 h after birth, it results in a left-to-right shunt through the ductus, causing an overload of the pulmonary circulation [1]. Hemodynamically significant patent ductus arteriosus (hsPDA) [2], as identified by echocardiographic criteria, exerts a more pronounced impact on preterm neonates, and which can lead to intestinal hypoperfusion and even necrotizing enterocolitis (NEC) [3]. It was reported that neonates with patent ductus arteriosus (PDA) or other congenital heart diseases (CHD) would exhibit a higher incidence of more severe NEC after applying a mathematical formula for risk assessment [4]. The presence of PDA has been correlated with severe NEC or mortality in neonates [5], and it is recognized as an independent risk factor for the development of NEC in very low birth weight (VLBW) infants, as demonstrated by a population-based study [6]. Furthermore, a systematic review and meta-analysis conducted in China has identified PDA as a significant risk factor for NEC in preterm neonates, with an odds ratio (OR) of 3.1 [7]. Early surgical ligation of PDA within the first 10 days of life has been shown to reduce the incidence of severe NEC in preterm neonates compared to those who undergo late ligation after 10 days of age [8].

Previous studies on NEC in neonates with PDA have focused on mortality due to NEC, the incidence of Bell stage III NEC, the necessity for surgery or hospitalization for 65 days in survivors and have noted that the presence of PDA is associated with an increased risk of death [9]. However, these studies have not provided a comprehensive analysis of the presence of PDA whether have influences on multi-system functions, including respiratory, cardiovascular, nervous, coagulation, and urinary systems within one month following the onset of NEC. Echocardiography has been utilized primarily for assessing cardiac function in preterm infants with PDA [10] or for evaluating the outcomes of PDA pharmacotherapy [11], without addressing the specific issue of NEC in neonates with PDA.

The purpose of our study was to conduct a systematic evaluation of the characteristics of NEC in relation to the presence or absence of PDA. Additionally, we aimed to describe echocardiography parameters to facilitate a comparative analysis between neonates with NO-PDA and those with PDA, to assess the alterations in left ventricular function during the periods of NEC.

## Patients and methods

### Study population

#### The criteria of inclusion and exclusion

Two groups’ neonates were included based on Bell's criteria [12]: the mild NEC group (comprising stages I to IIA) and the severe NEC group (compassing stages IIB to III). Neonates in each group was further divided into two subgroups: the NO-PDA subgroup and the PDA subgroup. The NEC timeline was segmented into three periods: (a) before NEC occurred, (b) during NEC occurrence, and (c) within one month after NEC cured.

Inclusion criteria: 1) Preterm neonates diagnosed with NEC stage I to III were included in neonatal intensive care unit (NICU) from January 2015 to July 2022. 2) Neonates with 5 min Apgar score > 3 and a pH > 7.20 in umbilical artery blood gas within 24 h after birth. Neonates with birth asphyxia included in the study had only mild asphyxia (Apgar score = 4 ~ 7), without any severe brain injury or other CHD, and they were all alive and discharged. 3) All neonates included in the PDA subgroups were diagnosed with hsPDA after 72 h post-birth. Only those subjects whose DA remained open with a diameter greater than 1.5 mm at the time of NEC occurrence were classified as the PDA subgroup, as opposed to the NO-PDA subgroup. The criteria for diagnosing hsPDA were as follows, with items ① and ② being mandatory and at least one or more of item ③ required to be met [13, 14]: ① Clinical Symptoms: Deterioration in respiratory status, characterized by shortness of breath, aspiration, or an increase in required oxygen concentration; ② Clinical Precordial Auscultation Signs: Presence of continuous or systolic murmurs at the upper left sternal border, hyperdynamic precordial impulse, tachycardia, bounding pulses, widened pulse pressure, hypotension, or signs of heart failure. ③ Echocardiographic examination: a. A moderate‐to‐large transductal diameter (PDA diameter greater than 1.5 mm), b. Evidence of pulmonary overcirculation (left atrium to aortic root ratio greater than 1.5 mm); c. Evidence of systemic hypoperfusion (absent or reversed diastolic flow in the postductal descending aorta or celiac trunk or middle cerebral artery); d. Or a diameter indexed to body weight greater than 1.4 mm/kg in preterm neonates ≤ 32 weeks [14]. ④ We obtained verbal informed consent from the mothers of all neonates included in the study, and we are committed to protecting the privacy of all participants involved.

Exclusion criteria: 1) Neonates with severe birth asphyxia (Apgar score = 0 ~ 3). 2) Other CHD aside from PDA or those with heart failure were excluded. 3) Neonates with congenital gastrointestinal malformations, intestinal perforation, intussusception, or inflammatory enteritis were excluded. 4) Neonates with autoimmune system diseases or leukemia were excluded. 5) Neonates with chromosomal abnormalities, inherited metabolic or genetic diseases were excluded. 6) Neonates whose parents refused to provide detailed data or were lost to follow-up until three months after birth were excluded, as shown in sfig 1.

### Demographic characteristics

Demographic characteristics of neonates' mothers were documented: maternal age, delivery mode (cesarean or vaginal), multiple pregnancies, premature rupture of membranes, turbid amniotic fluid, antepartum use of magnesium sulfate and dexamethasone, maternal pregnancy complications (e.g., gestational diabetes mellitus (GDM), autoimmune diseases, intrahepatic cholestasis of pregnancy, hypertensive disorders in pregnancy (HDP), thyroid dysfunction during pregnancy, and placental abruption), and positive cervical secretion culture results. Concurrently, characteristics of neonates were recorded: the NEC stage, gestational age (GA), birth weight (BW), ductal diameter relative to BW (mm), gender (male), small for gestational age (SGA), birth asphyxia, and fetal intrauterine distress, as detailed in Table 1.

**Table 1:** Demographic Characteristics of the NO-PDA and PDA Subgroups in neonates with NEC

Variable	NEC stage I-IIA		*P*	NEC stage IIB-III		*P*
	NO-PDA (n1 = 43)	PDA (n2 = 38)		NO-PDA (m1 = 25)	PDA (m2 = 29)	
Maternal characteristics						
Maternal age	30.2 ± 4.5	30.5 ± 3.9	0.845	31.8 ± 4.0	29.6 ± 4.9	0.079
Cesarean delivery	22 (51.2%)	23 (60.5%)	0.397	16 (64.0%)	14 (48.3%)	0.246
Multiple pregnancies	12 (27.9%)	11 (28.9%)	0.917	13 (52.0%)	12 (41.4%)	0.625
Premature rupture of membranes	14 (32.6%)	9 (23.7%)	0.377	8 (32.0%)	6 (20.7%)	0.344
Turbid amniotic fluid	12 (27.9%)	8 (21.1%)	0.475	8 (32.0%)	5 (17.2%)	0.206
Gestational Diabetes Mellitus	21 (48.8%)	14 (36.8%)	0.277	5 (20.0%)	6 (20.7%)	0.950
Autoimmune Disease	1 (2.3%)	5 (13.2%)	0.152	0 (0.0%)	1 (3.4%)	0.262
Intrahepatic Cholestasis of Pregnancy	2 (4.7%)	3 (7.9%)	0.545	0 (0.0%)	2 (6.9%)	0.110
Hypertensive Disorders in Pregnancy	5 (11.6%)	6 (15.8%)	0.585	5 (20.0%)	2 (6.9%)	0.153
Thyroid dysfunction during pregnancy	5 (11.6%)	3 (7.9%)	0.574	7 (28.0%)	2 (6.9%)	0.088
Placental abruption	11 (25.6%)	8 (21.1%)	0.631	7 (28.0%)	6 (20.7%)	0.531
Cervical secretion culture results (+)	6 (13.9%)	8 (21.1%)	0.399	5 (20.0%)	10 (34.9%)	0.236
Antepartum use of magnesium sulfate	23 (53.5%)	24 (63.2%)	0.379	16 (64.0%)	18 (62.1%)	0.884
Antepartum use of dexamethasone	38 (88.4%)	37 (97.4%)	0.123	23 (92.0%)	25 (86.2%)	0.499
Neonatal characteristics						
Gestational Age (week)	29.1 ± 2.3	30.0 ± 2.9	0.161	29.4 ± 2.6	29.8 ± 3.0	0.538
Birth Weight (kg)	1.308 ± 0.352	1.423 ± 0.574	0.278	1.173 ± 0.248	1.322 ± 0.487	0.156
Ductal Diameter relative to Birth Weight (mm)	1.66 ± 0.80	1.66 ± 0.58	0.985	1.88 ± 0.76	2.03 ± 0.87	0.491
Male	28 (65.1%)	21 (55.3%)	0.365	14 (56.0%)	19 (65.5%)	0.474
Birth asphyxia	9 (20.9%)	8 (21.1%)	0.989	4 (16.0%)	4 (13.8%)	0.820
Fetal intrauterine distress	3 (7.0%)	9 (23.7%)	0.035	3 (12.0%)	11 (37.9%)	0.030
Small for gestational age	4 (9.3%)	5 (13.2%)	0.582	10 (40.0%)	6 (20.7%)	0.121

### Neonates outcomes

The basic characteristics were recorded, including ductal diameter relative to BW (mm), postmenstrual gestational age (PGA), age at the onset of NEC (days), and weights at the onset of NEC (kg). Before NEC occurred, the vital signs were also recorded, including rectal temperature (℃), respiratory rate (RR), systolic blood pressure (SBP), diastolic blood pressure (DBP), heart rates (HR), and transcutaneous oxygen saturation (TcSO_2_). The use of medications (sodium citrate caffeine and ibuprofen) and acquiring red blood cell transfusions were recorded before NEC occurred. Abdominal findings were contained: ascites, red blood cells or pyocytes in ascites, pneumatosis intestinalis or portal venous gas detected by abdominal ultrasound and blood culture results in cases of sepsis.

Bronchopulmonary Dysplasia (BPD) is defined as the continued requirement of oxygen support for newborns beyond 28 days post-natal or beyond 36 weeks of corrected GA. Neonatal sepsis is characterized by clinical infectious symptoms and a positive blood culture. Heart failure is diagnosed when one or more of the following are present: liver enlargement (≥ 3 cm), galloping rhythm, overt pulmonary edema, or severe circulatory failure, and including ST-T segment depressions detected by electrocardiogram. Neurological outcomes were assessed by cerebral ultrasound and included diagnoses such as ventriculomegaly, subependymal cyst, periventricular leukomalacia (PVL), and intraventricular hemorrhage (IVH). The complications within the severe NEC group, following surgical versus conservative treatment post-NEC occurrence, were detailed in sTable 1 and included electrolyte imbalances, intestinal stenosis, severe sepsis, and recurrence of NEC.

### Laboratory parameters

When NEC appeared, routine blood indicators were recorded including white blood cells (WBC) count, percentage of neutrophils (NE%), absolute neutrophil counts (NE), absolute lymphocyte counts (LY), neutrophil-to-lymphocyte ratio (N/L), hemoglobin (HGB), platelet count (PLT) and C-reactive protein (CRP). Blood biochemical results were recorded including, calcium (Ca^2+^), magnesium (Mg^2+^), phosphorus (PHOS), alanine aminotransferase (ALT), aspartate aminotransferase (AST), alkaline phosphatase (ALP), creatine kinase MB (CKMB), creatinine (CRE), and blood urea nitrogen (BUN). The prothrombin time (PT), activated partial thromboplastin time (APTT), fibrinogen (Fib), D-dimer, and fibrinogen degradation product (FDP) were recorded for coagulation parameters analysis. Blood gas analyses were recorded during periods of b and c, which included pondus hydrogenii value (PH), partial pressure of oxygen (pO_2_), partial pressure of carbon dioxide (pCO_2_), buffer excess value (BE), and lactic acid (Lac).

### Ventilator therapy

Most neonates diagnosed with NEC exhibited symptoms such as apnea or alterations in respiratory rate and rhythm. Consequently, immediate ventilator therapy or oxygen support was necessary to sustain normal respiratory function. The methods of ventilator therapy employed, including mask oxygen inhalation and various ventilator therapy modes (e.g., high-frequency oscillatory ventilation (HFOV), synchronized intermittent mandatory ventilation (SIMV), nasal continuous positive airway pressure (NCPAP), and Bi-level positive airway pressure (BiPAP)), were documented. We calculated respiratory mechanics parameters, including the oxygenation index (OI), PaO2/FiO2 ratio (P/F ratio), and mean airway pressure (MAP), under different ventilator modes, as shown in Table 3.

$${\varvec{M}}{\varvec{A}}{\varvec{P}}\boldsymbol{ }({\varvec{S}}{\varvec{I}}{\varvec{M}}{\varvec{V}})={\varvec{k}}\times \frac{{\varvec{P}}{\varvec{I}}{\varvec{P}}\times {\varvec{T}}{\varvec{I}}+{\varvec{P}}{\varvec{E}}{\varvec{E}}{\varvec{P}}\times {\varvec{T}}{\varvec{E}}}{{\varvec{T}}{\varvec{I}}+{\varvec{T}}{\varvec{E}}}$$

K = 1(Constant pressure mode); TI, inspiration time; TE, expiratory time; PIP, peak inspiratory pressure; PEEP, positive end expiratory pressure.

$${\varvec{P}}/{\varvec{F}}=\frac{{\varvec{P}}{\varvec{a}}{\varvec{O}}2}{{\varvec{F}}{\varvec{i}}{\varvec{O}}2}$$

FIO_2_, fraction of inspired oxygen; PaO_2_, partial pressure of arterial oxygen.

$${\varvec{O}}{\varvec{I}}={\varvec{M}}{\varvec{A}}{\varvec{P}}\times \frac{{\varvec{F}}{\varvec{I}}{\varvec{O}}2}{{\varvec{P}}{\varvec{a}}{\varvec{O}}2}$$

FIO_2_, fraction of inspired oxygen; PaO_2_, partial pressure of arterial oxygen.

### Echocardiographic parameters

Echocardiography was conducted using the Vivid IQ machine (General Electric Healthcare ultrasound, Austria), with a 6S-RS Doppler probe frequency ranging from 2.0 to 7.0 MHz. Measurements were taken of the left ventricular internal diameter at end-diastole (LVIDd) and left ventricular internal diameter at systole (LVIDs). All echocardiography procedures were performed by ultrasound physicians specializing in echocardiographic diagnosis with over a decade of experience. Fractional shortening (FS) was calculated using the following formula, as shown in Table 4 and Fig. 2.

$${\varvec{F}}{\varvec{S}}\boldsymbol{ }\left(\boldsymbol{\%}\right)=\frac{{\varvec{L}}{\varvec{V}}{\varvec{I}}{\varvec{D}}{\varvec{d}}-{\varvec{L}}{\varvec{V}}{\varvec{I}}{\varvec{D}}{\varvec{s}}}{{\varvec{L}}{\varvec{V}}{\varvec{I}}{\varvec{D}}{\varvec{d}}}$$

We quantified left ventricular end-diastolic volume (EDV) and end-systolic volume (ESV), and the left ventricular ejection fraction (EF) was derived using the following formula:

$${\varvec{E}}{\varvec{F}}\boldsymbol{ }\left(\boldsymbol{\%}\right)=\frac{{\varvec{E}}{\varvec{D}}{\varvec{V}}-{\varvec{E}}{\varvec{S}}{\varvec{V}}}{{\varvec{E}}{\varvec{D}}{\varvec{V}}}$$

Tricuspid regurgitation and mitral regurgitation were measured using color doppler flow imaging (CDFI). The severity was graded as follows: ‘ + ’ for mild tricuspid regurgitation, ‘ + + ’ for middle tricuspid regurgitation, and ‘ + +  + ’ for severe tricuspid regurgitation. The diameters of DA and Patent Foramen Ovale (PFO) were also measured [15].

### Statistical analyses

**Fig. 1:**
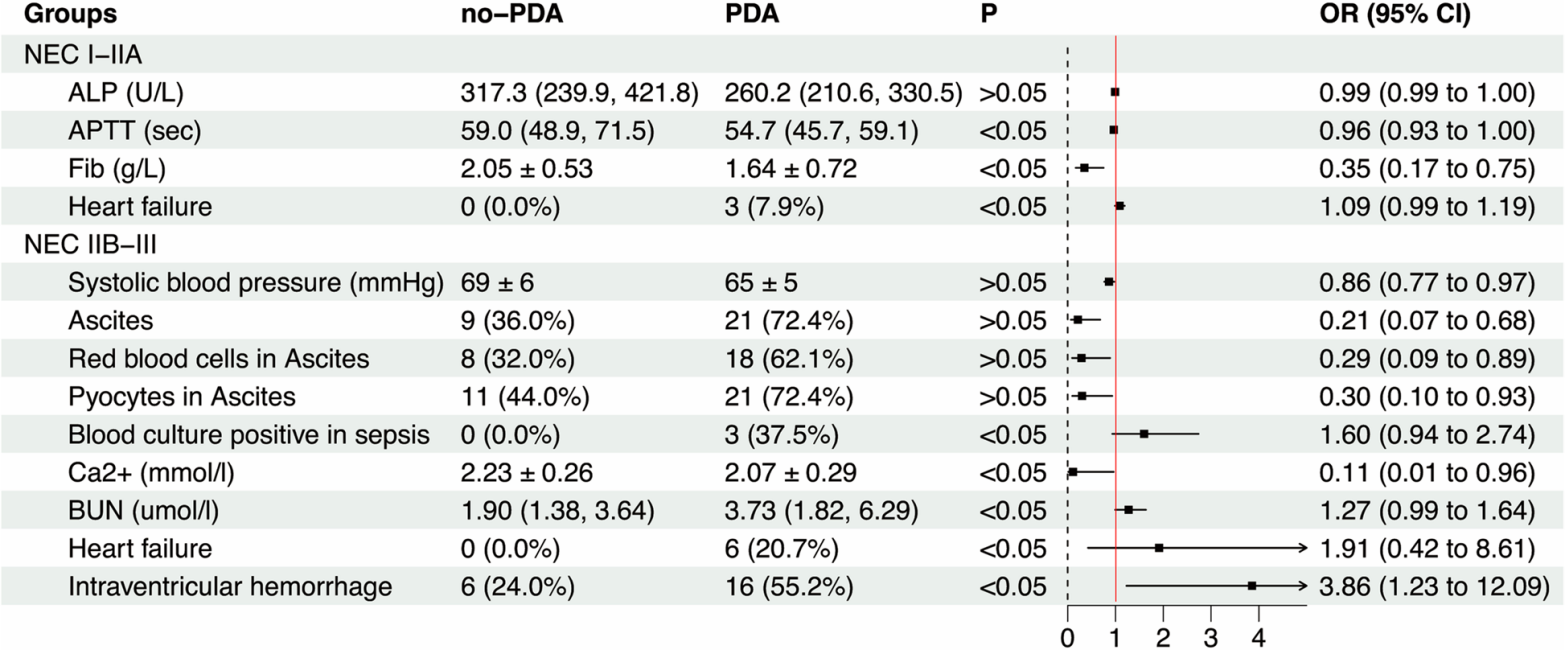
Comparative assessment of multi-system functions in neonates with NEC: NO-PDA Subgroup vs. PDA Subgroup (after adjustment)

The statistical analyses were performed using SPSS Statistics version 26.0 (IBM Corp, Armonk, NY, USA). The data were expressed as the mean ± SD (standard deviation) or the median (P_25_, P_75_) for continuous data and number (%) for categories variables. If fit to a normal distribution, the variables were analyzed by t-tests; if not, they were analyzed by the Mann–Whitney U test. Chi-square tests or Fisher’s exact tests were used in categorical variables. Unadjusted analysis results were presented in Table 2, while the adjusted results following multivariate logistic regression, with OR calculated, were shown in Fig. 1. This figure was generated utilizing the R programming language. *P* < *0.05* were considered statistically significant.

**Table 2:** Comparative assessment of multi-system functions and complications between the two subgroups in neonates with NEC (before adjustment)

Variable	NEC stage I-IIA		*P*	NEC stage IIB-III		*P*
	NO-PDA (n1 = 43)	PDA (n2 = 38)		NO-PDA (m1 = 25)	PDA (m2 = 29)	
Basic vital signs and drugs (a)						
Ductal Diameter relative to Birth Weight (mm)	0.00 ± 0.00	1.41 ± 0.56	< 0.001	0.04 ± 0.18	1.83 ± 0.77	< 0.001
PGA (weeks)	33.1 ± 2.1	33.5 ± 2.4	0.393	33.2 ± 2.7	32.3 ± 3.0	0.269
Age at the onset of NEC (days)	27.3 ± 13.8	25.2 ± 15.7	0.501	24.8 ± 10.4	19.5 ± 14.6	0.526
Weights at the onset of NEC (kg)	1.666 ± 0.267	1.771 ± 0.514	0.266	1.619 ± 0.366	1.448 ± 0.542	0.187
Anal temperature (℃)	36.9 (36.7, 37.1)	36.9 (36.8, 37.9)	0.580	37.0 (36.8, 37.6)	37.4 (36.8, 38.0)	0.151
Respiratory rate (times/min)	54 (49, 56)	54 (50, 56)	0.977	54 (50, 58)	55 (54, 61)	0.079
Systolic blood pressure (mmHg)	66 ± 5	67 ± 5	0.373	69 ± 6	65 ± 5	0.009
Diastolic blood pressure (mmHg)	35 ± 4	34 ± 4	0.142	34 ± 5	33 ± 4	0.537
Heart rates (times/min)	151 ± 11	154 ± 16	0.209	160 ± 23	154 ± 16	0.298
TcSO_2_ (%)	92 (90, 93)	93 (88, 94)	0.495	90 (80, 94)	92 (88, 93)	0.354
Sodium citrate caffeine	35 (81.4%)	27 (71.1%)	0.401	20 (80.0%)	22 (75.9%)	0.788
Ibuprofen	20 (46.5%)	12 (31.6%)	0.170	7 (28.0%)	13 (44.8%)	0.202
Red blood cell transfusions	15 (34.9%)	13 (34.2%)	0.949	7 (28.0%)	8 (27.6%)	0.973
Abdominal fingdings (b)						
Ascites	8 (18.6%)	6 (15.8%)	0.738	9 (36.0%)	21 (72.4%)	0.007
Red blood cells in Ascites	7 (16.3%)	4 (10.5%)	0.451	8 (32.0%)	18 (62.1%)	0.027
Pyocytes in Ascites	7 (16.3%)	6 (15.8%)	0.952	11 (44.0%)	21 (72.4%)	0.034
Pneumatosis intestinalis	10 (23.3%)	11 (28.9%)	0.560	6 (24.0%)	12 (41.4%)	0.378
Portal venous gas	0 (0.0%)	0 (0.0%)		16 (64.0%)	15 (51.7%)	
No abdominal ultrasound signs	33 (76.7%)	27 (71.1%)		3 (12.0%)	2 (6.9%)	
Blood culture results in cases of sepsis (+)/(-)	3/5	4/4	0.614	0/7	3/5	0.035
Blood routine and C-reactive protein results (b)						
WBC (× 10^9/L)	8.73 (5.45, 13.64)	8.34 (6.35, 13.42)	0.951	8.93 (6.51, 13.98)	8.23 (5.78, 11.56)	0.405
NE%	44.2 ± 18.1	45.3 ± 20.2	0.799	50.5 ± 20.3	52.0 ± 20.0	0.783
NE (× 10^9/L)	3.22 (1.66, 7.57)	3.60 (1.74, 8.07)	0.966	3.93 (2.46, 8.02)	3.40 (2.01,6.68)	0.395
LY (× 10^9/L)	3.23 (2.09, 4.79)	3.07 (2.38, 3.84)	0.674	3.20 ± 1.69	2.53 ± 1.43	0.121
N/L	1.1 (0.6, 2.4)	1.0 (0.6, 3.3)	0.970	1.4 (0.8, 3.4)	1.9 (1.0, 3.6)	0.664
HGB (g/L)	104.7 ± 20.7	111.9 ± 23.1	0.139	108.8 ± 21.4	110.6 ± 20.6	0.780
PLT (× 10^9/L)	290.8 ± 17.3	290.2 ± 18.3	0.751	300.1 ± 26.9	232.6 ± 28.1	0.091
CRP (mg/dl)	0.93 (0.50, 5.21)	2.04 (0.64, 5.21)	0.316	1.04 (0.50, 3.00)	3.00 (1.20, 26.17)	0.051
Blood Biochemical results (b)						
Ca^2+^ (mmol/l)	2.29 ± 0.20	2.27 ± 0.14	0.629	2.23 ± 0.26	2.07 ± 0.29	0.025
Mg^2+^ (mmol/l)	0.81 (0.71, 0.86)	0.83 (0.75, 0.88)	0.210	0.79 ± 0.19	0.74 ± 0.18	0.304
PHOS (mmol/l)	1.71 ± 0.38	1.69 ± 0.32	0.798	1.81 ± 0.40	1.61 ± 0.35	0.051
ALT (U/L)	5.14 (3.10, 7.64)	6.53 (4.20, 9.75)	0.069	5.20 (3.45, 9.80)	6.13 (4.48, 9.14)	0.381
AST (U/L)	18.5 (15.3, 24.5)	21.2 (18.1, 27.5)	0.068	22.1 (18.4, 30.0)	27.91 (8.1, 35.4)	0.420
ALP (U/L)	317.3 (239.9, 421.8)	260.2 (210.6, 330.5)	0.031	322.3 (236.5, 474.9)	219.2 (182.3, 340.8)	0.051
CKMB (U/L)	35.2 (28.3, 63.1)	38.4 (29.3, 59.0)	0.925	42.2 (26.7, 91.5)	44.4 (36.1, 56.2)	0.768
CRE (umol/l)	40.5 (36.4, 46.4)	42.2 (33.2, 47.5)	0.755	40.2 (35.0, 50.4)	44.4 (32.8, 57.9)	0.510
BUN (umol/l)	2.62 (1.69, 4.26)	2.24 (1.52, 3.36)	0.344	1.90 (1.38, 3.64)	3.73 (1.82, 6.29)	0.044
Coagulation parameters results (b)						
PT (sec)	14.3 (13.1, 15.8)	14.4 (13.2, 15.4)	0.687	14.6 (13.4, 16.1)	15.4 (13.6, 18.3)	0.129
APTT (sec)	59.0 (48.9, 71.5)	54.7 (45.7, 59.1)	0.018	54.3 (46.2, 67.9)	56.7 (47.6, 74.7)	0.267
Fib (g/L)	2.05 ± 0.53	1.64 ± 0.72	0.005	1.93 ± 1.09	1.92 ± 0.74	0.549
FDP (mg/L)	3.66 (2.47, 7.30)	5.29 (3.21, 6.94)	0.285	5.61 (3.82, 9.50)	5.66 (3.58, 18.23)	0.742
D-dimer(g/L)	0.98 (0.76, 1.92)	1.58 (0.96, 2.30)	0.083	1.11 (0.85, 2.49)	2.14 (0.93, 5.09)	0.117
Neonatal complications (c)						
Bronchopulmonary dysplasia	6 (14.0%)	8 (21.1%)	0.399	6 (24.0%)	5 (17.2%)	0.539
Neonatal sepsis	5 (11.6%)	4 (10.5%)	0.875	4 (16.0%)	5 (17.2%)	0.903
Heart failure	0 (0.0%)	3 (7.9%)	0.031	0 (0.0%)	6 (20.7%)	0.004
ST—T segment depression	4 (9.3%)	8 (21.1%)	0.137	14 (56.0%)	13 (44.8%)	0.413
Ventriculomegaly	25 (58.1%)	20 (52.6%)	0.619	13 (52.0%)	20 (69.0%)	0.202
Subependymal cyst	8 (18.6%)	8 (21.1%)	0.782	6 (24.0%)	7 (24.1%)	0.991
Periventricular leukomalacia	1 (2.3%)	0 (0.0%)	0.344	0 (0.0%)	1 (3.4%)	0.349
Intraventricular hemorrhage	10 (23.3%)	14 (36.8%)	0.181	6 (24.0%)	16 (55.2%)	0.020

PGA**,** Postmenstrual gestational age; TcSO_2_, Transcutaneous oxygen saturation; WBC, white blood cells; NE%, percentage of neutrophils; NE, neutrophil count; LY, lymphocyte count; N/L, neutrophil-to-lymphocyte ratio; HGB, hemoglobin; PLT, platelet count; CRP, C-reactive protein; Ca^2+^, calcium; Mg^2+^, magnesium; PHOS, phosphorus; ALT, alanine aminotransferase; AST, aspartate aminotransferase; ALP, alkaline phosphatase; CKMB, creatine kinase MB; CRE, creatinine; BUN, blood urea nitrogen; PT, prothrombin time; APTT, activated partial thromboplastin time; Fib, fibrinogen; FDP, fibrinogen degradation products. a: before NEC occurred; b: during NEC occurrence; c: within one month after NEC cured

## Results

### Study population

As the largest neonatal rescue center on the east-south coast of China, our hospital has seen over 20,000 deliveries and admits more than 2,000 preterm neonates annually. The incidence rate of NEC in our NICU over the span of 7 years was 4.99%, and the mortality rate was significantly lower than that reported in the literature [16]. Initially, 589 preterm neonates were identified from January 2015 to July 2022, and it was found that 63.8% (376/589) of these neonates had the presence of PDA when NEC occurred. After the exclusion process, a total of 135 preterm neonates with a GA ranging from 24^+6^ to 36^+4^ weeks were ultimately enrolled in the study, including 46.9% (38/81) of neonates in the mild NEC group and 53.7% (29/54) of neonates in the severe NEC group.

The neonates were subsequently categorized into two groups: the mild NEC group (*n* = 81) and the severe NEC group (*n* = 54). Each group was further divided into two subgroups: the NO-PDA subgroup (n1 = 43 for the mild NEC group, m1 = 25 for the severe NEC group) and the PDA subgroups (n2 = 38 for the mild NEC group, m2 = 29 for the severe NEC group), as shown in sFig 1 and tables. In the mild NEC group, the average GA and BW were 29.1 ± 2.3 weeks and 1.308 ± 0.352 kg for the NO-PDA subgroup, and 30.0 ± 2.9 weeks and 1.423 ± 0.574 kg for the PDA subgroup, respectively. In the severe NEC group, the average GA and BW were 29.4 ± 2.6 weeks and 1.173 ± 0.248 kg for the NO-PDA subgroup, and 29.8 ± 3.0 weeks and 1.322 ± 0.487 kg for the PDA subgroup, respectively. Maternal and neonatal characteristics did not differ significantly between the two subgroups, except for fetal intrauterine distress (*P* = *0.03*), as shown in Table 1. There were no initial differences in ductal diameter relative to BW between hsPDA neonates in the NO-PDA subgroup and PDA subgroup (*P* > *0.05*), as shown in Table 1. However, during the occurrence of NEC, neonates in the PDA subgroup still presented with a PDA and a DA diameter exceeding 1.5 mm, in contrast to those in the NO-PDA subgroup who did not (*P* < 0.001), as illustrated in Table 2.

### Neonates outcomes

In the mild NEC group, the levels of ALP, APTT, and Fib were significantly lower in the PDA subgroup compared to the NO-PDA subgroup (*P* < *0.05*), as shown in Table 2. In the severe NEC group, 92.1% of neonates had a closed DA, compared to only 79.3% in the mild NEC group during period c. After adjustment, for every one gram per liter decrease in fibrinogen, the risk of NEC occurrence increased by 65% (*P* < *0.05*), as shown in Fig. 1. Neonates in the severe-NEC group who underwent conservative treatment were more likely to experience electrolyte imbalances than those who received surgical treatment (*P* = *0.01*), as detailed in sTable 1.

Before adjustment, in the severe NEC group, the SBPs of neonates with PDA were lower than those of neonates without PDA *(P* = *0.009*). During period b, the PDA subgroup had a higher incidence of ascites, presence of red blood cells or pyocytes in ascites, and positive blood culture results (*P* < *0.05*). The Ca2 + levels in the PDA subgroup were lower, and the BUN levels were higher compared to the NO-PDA subgroup (*P* < *0.05*). Heart failure and IVH were the main complications differing between the two subgroups (*P* < *0.05*), as shown in Table 2. After adjustment, the occurrence of increased BUN, positive blood culture results in cases of sepsis, heart failure, and IVH were 1.27, 1.60, 1.91, and 3.86 times higher in the PDA subgroup compared to the NO-PDA subgroup (*P* < *0.05*), respectively. Additionally, for each one mmol/l decrease in Ca2 + levels, the risk of severe NEC incidence increased by 89% in neonates with PDA (*P* < *0.05*), as shown in Fig. 1.

### Ventilator therapy

The levels of pCO2 during period b and the utilization of ventilator support were significantly higher in the PDA subgroup of the severe NEC group *(P* < *0.05*). Lower P/F ratios under mask oxygen inhalation were observed in neonates with PDA across both the mild and severe NEC groups (*P* < *0.05*). The OI-1 for SIMV, OI-2 for HFOV, and MAP-2 for HFOV in the PDA subgroup were poorer compared to those in the NO-PDA subgroup of the severe NEC group (*P* < *0.05*), as detailed in Table 3.

**Table 3:** Comparative assessment of ventilator therapy between the NO-PDA and PDA subgroups in neonates with NEC

Variables	NEC stage I-IIA		P	NEC stage IIB-III		P
	NO-PDA (n1 = 43)	PDA (n2 = 38)		NO-PDA (m1 = 25)	PDA (m2 = 29)	
Blood gas analysis (b, c)						
pO_2_-b	79.5 ± 22.6	74.4 ± 23.6	0.235	80.2 ± 23.3	73.7 ± 25.7	0.333
pCO_2_-b	42.3 ± 10.2	43.6 ± 11.8	0.583	39.6 ± 6.4	46.7 ± 13.4	0.012
PH-b	7.37 ± 0.07	7.37 ± 0.07	0.935	7.36 ± 0.10	7.32 ± 0.10	0.138
BE-b	−1.05 ± 0.49	−0.32 ± 0.78	0.431	−1.82 ± 1.02	−2.34 ± 0.83	0.749
Lac-b	1.6 ± 0.1	1.5 ± 0.1	0.605	2.8 ± 0.8	2.3 ± 0.6	0.699
pO_2_-c	78.6 ± 26.2	73.3 ± 25.7	0.272	81.3 ± 25.3	69.7 ± 30.4	0.138
pCO_2_-c	42.8 ± 8.7	42.2 ± 10.7	0.779	44.0 ± 11.4	47.1 ± 17.4	0.442
PH-c	7.39 ± 0.06	7.37 ± 0.07	0.194	7.36 ± 0.08	7.33 ± 0.06	0.434
BE–c	−0.13 ± 0.38	0.03 ± 0.67	0.834	0.10 ± 0.53	−2.17 ± 0.79	0.003
Lac-c	1.3 ± 0.1	1.6 ± 0.2	0.338	1.9 ± 0.3	2.6 ± 0.6	0.781
Ventilator therapy parameters (b, c)						
Ventilator support-b	18/25	18/20	0.619	8/17	20/9	0.007
Ventilator support-c	24/19	19/19	0.601	17/8	24/5	0.206
Invasive/non-invasive ventilator	11/13	11/8	0.432	9/8	19/5	0.075
Invasive ventilator mode (HFOV/SIMV)	2/9	5/6	0.360	2/7	5/14	0.815
Non-invasive ventilator mode (NCPAP/BiPAP)	2/11	3/5	0.530	1/7	3/2	0.235
Respiratory Mechanics parameters (c)						
P/F ratio (Mask)	274.0 ± 96.1	221.4 ± 116.6	0.043	318.5 ± 146.7	194.2 ± 98.4	0.002
OI-1 (SIMV)	2.2 (1.5, 5.1)	2.6 (2.3, 4.6)	0.343	2.3 (1.4, 3.0)	3.6 (2.9, 5.0)	0.017
OI-2 (HFOV)	3.1 (2.8, 3.4)	8.9 (4.9, 13.6)	0.079	8.1 (6.8, 9.3)	16.7 (15.8, 18.8)	0.012
MAP-1 (SIMV)	5.2 (4.8, 9.3)	7.0 (4.8, 8.6)	0.849	7.8 (4.8, 9.0)	8.8 (7.4, 9.5)	0.165
MAP-2 (HFOV)	10.0 (8.0, 12.0)	10.0 (8.3, 12.5)	0.693	11.5 (11.0, 12.0)	14.0 (13.0, 14.0)	0.046

pO_2_, partial pressure of oxygen; pCO_2_, partial pressure of carbon dioxide; PH, pondus hydrogenii value; BE, buffer excess value; Lac, lactic acid; HFOV, high-frequency oscillatory ventilation; SIMV, Synchronized Intermittent Mandatory Ventilation; NCPAP, Nasal Continuous Positive Airway Pressure; BiPAP, Bi-level Positive Airway Pressure; P/F ratio, PaO₂/FiO₂ ratio; OI, Oxygenation index; MAP, Mean airway pressure. a: before NEC occurred; b: during NEC occurrence; c: within one month after NEC cured

### Echocardiographic parameters

**Fig. 2:**
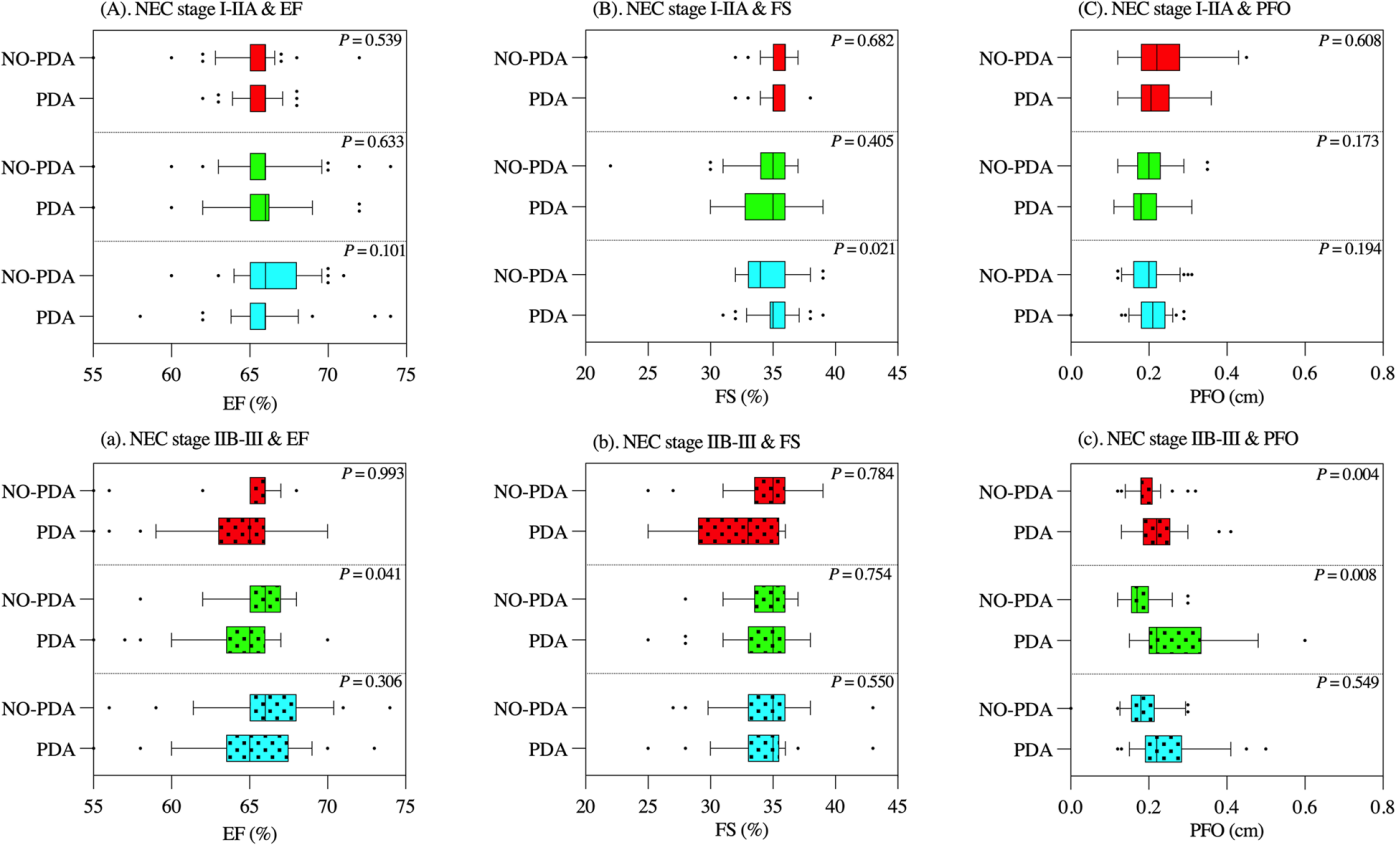
The comparison of left ventricular systolic function and diameters of patent foramen ovale (PFO) between the NO-PDA and PDA subgroups during the NEC periods

Table 4 was designed to analyze whether there were different echocardiographic parameters when neonates had the presence of PDA at various stages of NEC, comparing the mild and severe NEC groups. Figure 2 was intended to elucidate whether cardiac function and the diameters of PFO in neonates with PDA differed within the same stage and period of NEC compared to those without PDA. Tricuspid regurgitation, rather than mitral regurgitation, showed a significant difference between the two groups during the NEC process (*P* < *0.05*). In period a, the EF and FS were lower in the severe NEC group than in the mild NEC group (*P* < *0.05*). During period b, the PFO diameter (cm) was larger in the severe NEC group than in the mild NEC group (*P* < *0.05*), as shown in Table 4. In the severe NEC group, EF in the PDA subgroup was significantly lower than in the NO-PDA subgroup (*P* = *0.041*), as shown in Fig. 2. In period c, there was a difference in FS between the mild and severe NEC groups (*P* = *0.013*), as shown in Table 4. There were found significant differences in FS between the two subgroups in the mild NEC groups during period c (*P* = *0.021*), as shown in Fig. 2. The PFO diameters in the PDA subgroups were larger than those in the NO-PDA subgroups during periods a and b (*P* < *0.05*), as shown in Fig. 2.

**Table 4:** Echocardiographic parameters of neonates with PDA across various stages of NEC

Variables	NEC stage I-IIA	NEC stage IIB-III	*P*
	(*n* = 38)	(m = 29)	
before NEC occurred (a)			
Diameter of DA-a (cm)	0.21 ± 0.07	0.24 ± 0.08	0.214
Direction of blood flow at the PDA-a			
left to right shunt	37 (97.4%)	26 (89.7%)	0.424
Bi-directional shunt	1 (2.6%)	3 (10.3%)	
EF-a (%)	65.7 ± 1.2	64.2 ± 3.6	0.018
FS-a (%)	35.2 ± 1.3	32.1 ± 3.3	< 0.01
Tricuspid regurgitation-a (+ + ~ + + +)	13 (34.2%)	18 (62.1%)	0.023
Mitral regurgitation-a (+ + ~ + + +)	3 (7.9%)	5 (17.2%)	0.430
Diameter of PFO-a (cm)	0.22 ± 0.05	0.23 ± 0.06	0.685
during NEC occurrence (b)			
Diameter of DA-b (cm)	0.18 ± 0.07	0.20 ± 0.10	0.111
Direction of blood flow at the PDA-b			
left to right shunt	37 (97.4%)	26 (89.7%)	0.424
Bi-directional shunt	1 (2.6%)	3 (10.3%)	
EF-b (%)	65.7 ± 3.0	64.2 ± 3.6	0.081
FS-b (%)	34.3 ± 2.1	33.8 ± 2.8	0.995
Tricuspid regurgitation-b (+ + ~ + + +)	9 (23.7%)	19 (65.5%)	0.001
Mitral regurgitation-b (+ + ~ + + +)	3 (7.9%)	6 (20.7%)	0.128
Diameter of PFO-b (cm)	0.19 ± 0.04	0.28 ± 0.11	< 0.01
within one month after NEC cured (c)			
DA diameter-c (cm)	0.06 ± 0.10	0.11 ± 0.16	0.156
Direction of blood flow at the PDA-c			
left to right shunt	11 (28.9%)	10 (34.5%)	0.044
Bi-directional shunt	0 (0.0%)	3 (10.3%)	
EF-c (%)	65.8 ± 2.6	65.1 ± 4.0	0.908
FS-c (%)	35.3 ± 1.6	33.9 ± 3.2	0.013
Tricuspid regurgitation-c(+ + ~ + + +)	1 (2.6%)	9 (31.0%)	0.004
Mitral regurgitation-c(+ + ~ + + +)	1 (2.6%)	4 (13.8%)	0.210
Diameter of PFO-c (cm)	0.21 ± 0.05	0.24 ± 0.10	0.261

PDA, patent ductus arteriosus; DA, ductus arteriosus; EF, ejection fraction; FS, shortening fraction; PFO, patent foramen ovale

## Discussions

Despite the fact that there is still a certain likelihood of DA closure after birth, even in premature neonates [17], numerous factors can contribute to prolonged DA patency. Neonates with PDA had a higher incidence of fetal intrauterine distress, as hypoxic factors could lead to the persistent patency of the arterial catheter after birth [18, 19].

### PDA impact on NEC

A reduction in systemic blood flow can lead to decreased mesenteric perfusion. Our findings indicated that because of the presence of PDA, a moderate to severe tricuspid regurgitation (+ +  ~ +  + +) was more likely to occur in the severe NEC group, potentially due to increased pulmonary vascular resistance and reduced blood flow to the left upper and lower body could finally lead to the occurrence of severe NEC [20]. In a premature baboon model, a moderate PDA shunt was found to impair the ability to increase postprandial superior mesenteric blood flow velocities and to reduce vascular resistance [21]. Ascites, red blood cells in ascites, and pyocytes in ascites were more likely to be detected in the PDA subgroup, which had relatively more severe intestinal lesions, poorer mesenteric blood flow volume supply, and more severe damage to the mesenteric barrier. The positive blood culture results in cases of sepsis for the PDA subgroup was 1.6 times higher than those of the NO-PDA subgroup, possibly due to perinatal infections that negatively impact the pharmacological closure of the ductus and increase the need for a second course of treatment or surgery in preterm neonates [22]. The presence of PDA could increase the incidence of gastrointestinal perforation due to decreased organ blood flow caused by ductal steal [23]. Although it is reported that perturbations in mesenteric blood flow, known as “diastolic steal,” contribute to intestinal hypoperfusion in neonates with PDA across NEC [21], medications used for PDA closure can decrease mesenteric blood flow. A large cohort study showed that therapy with indomethacin did not significantly affect the risk for NEC in the presence of PDA [24]. Ibuprofen, as a COX-inhibitor, not only retains similar efficacy to indomethacin but also has been shown to not cause NEC due to its less pronounced effects on intestinal hemodynamics and is therefore recommended for the treatment of PDA more recently [25].

### NEC neonates with PDA impact on Respiratory system

**Fig. 3:**
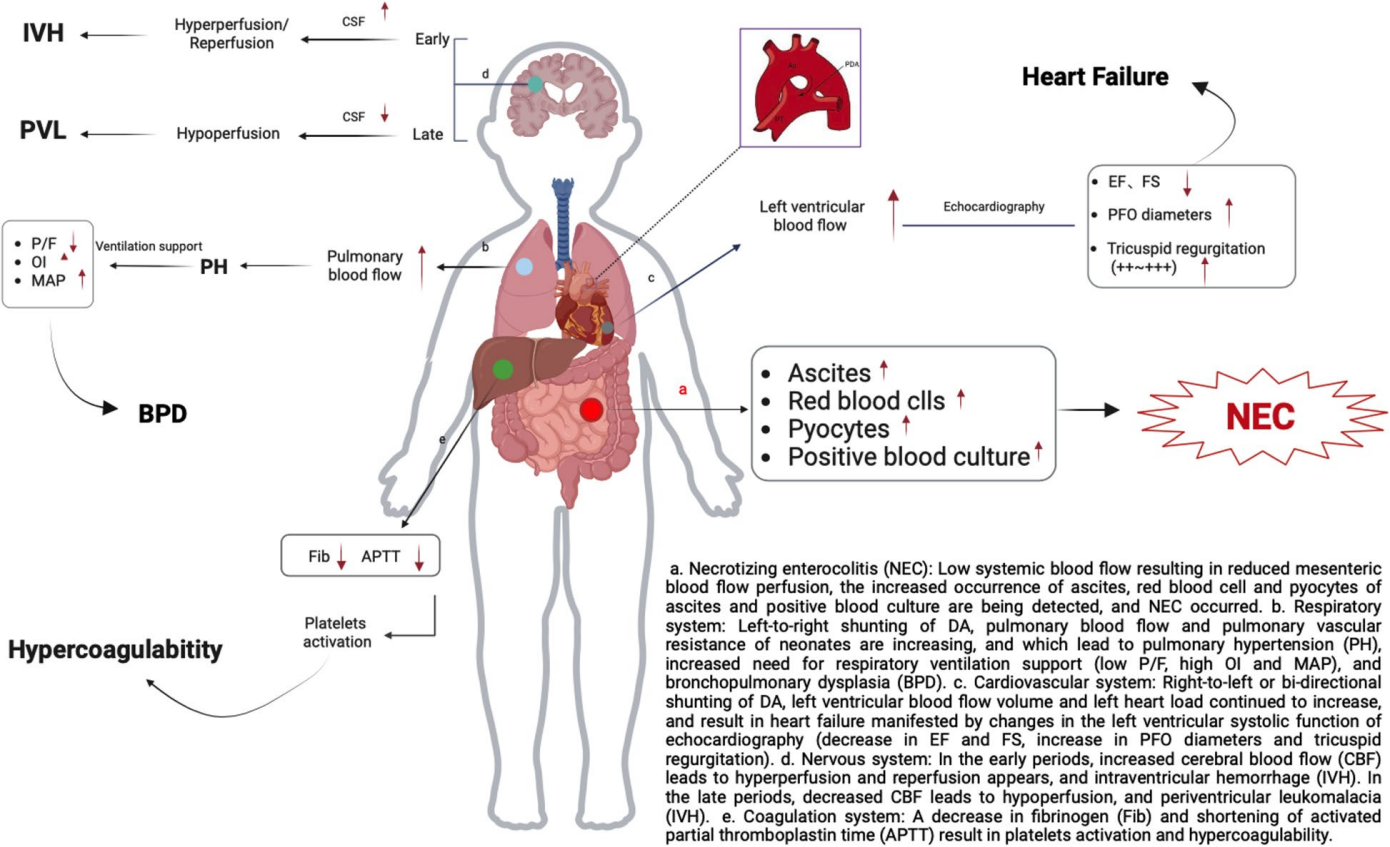
The presence of PDA exerts significant effects on preterm neonates with NEC

Elevated pCO2 and decreased BE, indicative of respiratory acidosis, along with a greater need for ventilator therapy, were more prevalent in the PDA subgroup. Large and continuous ductal shunting, which increases pulmonary blood flow, negatively impacts lung function, including the need for heightened respiratory support and susceptibility to lung diseases. The presence of a clinically detectable PDA has been noted prior to or at the time of pulmonary hemorrhage [26]. Moderate-to-large PDAs, along with intubation requirements of ≥ 10 days, were associated with increased risks of BPD, but not when intubation was required for less than 10 days [27]. We did not observe an increased occurrence of BPD in the PDA subgroup, as only 2 out of 135 neonates required intubation for more than 10 days. However, lower P/F ratios, higher OI and MAP requirements were noted in neonates with PDA when severe NEC occurred, as shown in Fig. 3. In VLBWs, a lower OI was associated with a higher incidence of PDA closure and fewer endotracheal intubations [28]. Infants with hsPDA exhibit lower dynamic lung compliance, higher pulmonary resistance, and require respiratory support with higher MAP [29, 30]. Conversely, a lower P/F ratio indicates a poorer outcome for lung diseases [31], and we also found a lower P/F ratio in the PDA subgroup of neonates with NEC.

### NEC neonates with PDA impact on cardiovascular system

The left ventricular blood flow volume continues to increase, leading to an increased left heart load and affecting left ventricular systolic function [32]. Decreased left ventricular systolic function was observed in the neonates with PDA either in the mild nor severe NEC group, as shown in Fig. 2. Neonates in the PDA subgroup had lower SBP and larger diameters of PFO within the severe NEC group (Table 2), because, despite the distensible PFO potentially reducing excessive right-to-left shunting from the DA in the left ventricle, it still could not prevent a decrease in systemic blood flow and post-ductal hypotension [33]. Acute right heart failure was more likely to occur in neonates with PDA (OR = 1.91). When this happened, Cedilanid was administered intravenously, either through rapid saturation or slow saturation.

### NEC neonates with PDA impact on nervous system

In the context of hsPDA, alterations in cerebral blood flow (CBF) have been recognized, particularly in cases of severe IVH, and larger early PDA shunts and lower right ventricular output may support a hypoperfusion or reperfusion etiological link [34]. We observed that the occurrence of IVH in the PDA subgroup was 3.86 times higher than that in the other subgroup of the severe NEC group. Studies have reported a higher incidence of grade ≥ 3 IVH in severe NEC cases associated with the presence of PDA [35]. Due to the presence of PDA, lower systemic perfusion followed by an increase in CBF precedes the development of IVH [36]. Although we did not find a higher incidence rate of PVL in neonates in our study, possibly due to a lack of sufficient long-term follow-up, the development of PVL has been proposed as a hemodynamic consequence associated with neurodevelopmental impairment. One possible mechanism leading to PVL in neonates with PDA may involve prolonged exposure to increased flow, leading to cerebral vascular remodeling, subsequent tissue ischemia, and the development of absent or reversed end-diastolic flow, exacerbating cerebral injury [37].

### NEC neonates with PDA impact on other system

For every one gram per liter decrease in Fib, the incidence risk of NEC increased by 65%. Meanwhile, a shorter APTT indicated platelet activation when the DA was patent in neonates with NEC. Decreased levels of Fib, and increased levels of fibronectin protein may heighten the risk of PDA [38].

For each one mmol/l decrease in Ca^2+^ levels, the incidence risk of NEC (IIB-III) increased by 89% in neonates with PDA. Higher Ca^2+^ levels are associated with spontaneous closure of the PDA, due to the role of calcium sensitization during the normoxic contraction of the DA [39]. A significantly higher mean BUN level was also observed with ibuprofen treatment [40], which aligns with our study findings.

### Limitations and expectation

This study represents the first to systematically analyze the multi-system functions and characteristics of NEC in relation to the presence or absence of PDA. Although we conducted a comparison between NO-PDA and PDA neonates to assess differences in left ventricular function using echocardiographic parameters, we were unable to obtain more detailed parameters regarding the diastolic function of the left or right ventricle due to the constraints of bedside ultrasound examinations. Furthermore, long-term follow-up of outcomes for preterm neonates with PDA is essential, and we are committed to tracking these outcomes continuously.

## Conclusions

HsPDA in neonates not only affects the severity and progression of NEC but also exerts an impact on multi-system functions. This includes exacerbating respiratory issues (due to high pulmonary blood flow and increased need for ventilatory support), increasing the load on the left ventricular blood flow, promoting hypercoagulability, disrupting calcium metabolism, and raising the risk of sepsis and IVH.

## Supplementary Information

Supplementary Material 1.

Supplementary Material 2.

## Data Availability

All data analyzed during this study are included in this published article and its supplementary information file. The datasets analyzed during the current study are available from the corresponding author on reasonable request.

## Abbreviations

ALT: Alanine aminotransferase
AST: Aspartate aminotransferase
ALP: Alkaline phosphatase
APTT: Activated partial thromboplastin time
BUN: Blood urea nitrogen
BE: Buffer excess value
BW: Birth weight
BiPAP: Bi-level positive airway pressure
BPD: Bronchopulmonary dysplasia
Ca^2+^: Calcium
CRP: C-reactive protein
CHD: Congenital heart disease
CDFI: Color doppler flow imaging
CKMB: Creatine kinase MB
CRE: Creatinine
DA: Ductus arteriosus
DBP: Diastolic blood pressure
EF: Ejection fraction
EDV: End-diastolic volume
ESV: End-systolic volume
ELBW: Extremely low birth weight
FS: Fractional shortening
Fib: Fibrinogen
FDP: Fibrinogen degradation product
GA: Gestational age
GDM: Gestational diabetes mellitus
HGB: Hemoglobin
HR: Heart rates
HDP: Hypertensive disorders in pregnancy
HFOV: High-frequency oscillatory ventilation
HsPDA: Hemodynamically significant patent ductus arteriosus
LY: Lymphocyte
Lac: Lactic acid
LVIDd: Left ventricular internal diameter at end-diastole
LVIDs: Left ventricular internal diameter at systole
Mg^2+^: Magnesium
MAP: Mean airway pressure
NCPAP: Nasal continuous positive airway pressure
N/L: Neutrophil-to-lymphocyte ratio
NE%: Percentage of neutrophils
NE: Absolute neutrophil
NICU: Neonatal intensive care unit
NEC: Necrotizing enterocolitis
OR: Odds ratio
OI: Oxygenation index
PH: Pondus hydrogenii value
pO_2_: Partial pressure of oxygen
pCO_2_: Partial pressure of carbon dioxide
PT: Prothrombin time
PFO: Patent foramen ovale
P/F ratio: PaO₂/FiO₂ ratio
PHOS: Phosphorus
PLT: Platelet count
PDA: Patent ductus arteriosus
PGA: Postmenstrual gestational age
PVL: Periventricular leukomalacia
RR: Respiratory rate
SIMV: Synchronized intermittent mandatory ventilation
SBP: Systolic blood pressure
SGA: Small for gestational age
TcSO_2_: Transcutaneous oxygen saturation
WBC: White blood cells
