# Supplementary figures and images for: Characteristics of neonatal necrotizing enterocolitis in relation to the presence or absence of patent ductus arteriosus
Source: BMC Pregnancy Childbirth. 2025 Jun 2;25:642. doi: 10.1186/s12884-025-07721-x (PMC12128525; doi:10.1186/s12884-025-07721-x)

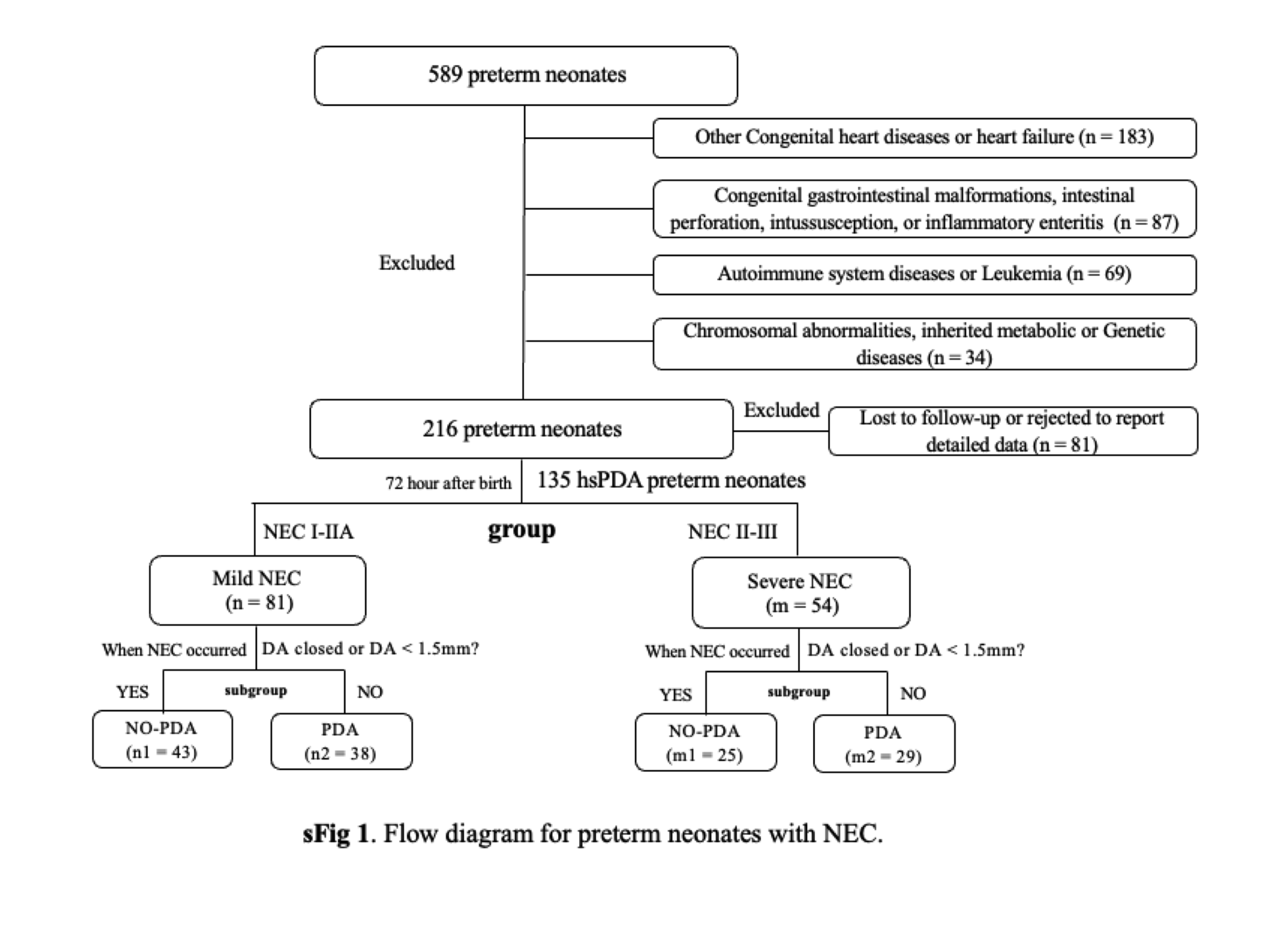

Supplement: Supplementary file 1 — Supplementary Material 1. [file 12884_2025_7721_MOESM1_ESM.tiff]
