# Supplementary material for: Characteristics of neonatal necrotizing enterocolitis in relation to the presence or absence of patent ductus arteriosus
Source: BMC Pregnancy Childbirth. 2025 Jun 2;25:642. doi: 10.1186/s12884-025-07721-x (PMC12128525; doi:10.1186/s12884-025-07721-x)
Supplement: Supplementary file 2 — Supplementary Material 2. [file 12884_2025_7721_MOESM2_ESM.docx]

| **sTable 1.** Complications in the Severe NEC Group: Surgical vs. Conservative Treatment after NEC cured. | | | |
| --- | --- | --- | --- |
| Variable | Conservative treatment | Surgical treatment | *P* |
|  | (31) | (23) |  |
| Electrolyte imbalance | 19 (61.3%) | 6 (26.1%) | 0.010 |
| Intestinal stenosis | 7 (22.6%) | 3 (13.0%) | 0.591 |
| Severe sepsis | 6 (19.4%) | 9 (39.1%) | 0.109 |
| recurrence of NEC | 1 (3.2%) | 1 (4.3%) | 0.829 |
